# Updates on the WHO diagnosis of IDH-mutant glioma

**DOI:** 10.1007/s11060-023-04250-5

**Published:** 2023-01-30

**Authors:** David.E. Reuss

**Affiliations:** 1 Department of Neuropathology, Institute of Pathology, Heidelberg University Hospital, Heidelberg, Germany; 2 Clinical Cooperation Unit Neuropathology, German Cancer Research Center (DKFZ), German Consortium for Translational Cancer Research (DKTK), Heidelberg, Germany

**Keywords:** WHO, Glioma, Classification, CNS5, Molecular pathology, Neuropathology, IDH-mutant

## Abstract

**Purpose:**

The WHO classification of Tumors of the Central Nervous System represents the international standard classification for brain tumors. In 2021 the 5th edition (WHO CNS5) was published, and this review summarizes the changes regarding IDH-mutant gliomas and discusses unsolved issues and future perspectives.

**Methods:**

This review is based on the 5th edition of the WHO Blue Book of CNS tumors (WHO CNS5) and relevant related papers.

**Results:**

Major changes include taxonomy and nomenclature of IDH-mutant gliomas. Essential and desirable criteria for classification were established considering technical developments. For the first time molecular features are not only relevant for the classification of IDH-mutant gliomas but may impact grading as well.

**Conclusion:**

WHO CNS5 classification moves forward towards a classification which is founded on tumor biology and serves clinical needs. The rapidly increasing knowledge on the molecular landscape of IDH-mutant gliomas is expected to further refine classification and grading in the future.

## Introduction

The WHO Classification of Tumors of the Central Nervous System from 2021 represents the 5th edition (WHO CNS5) of the international standard classification for brain tumors [1]. WHO CNS5 is a further development along the lines of the 2016 update of the 4th edition by reinforcing the role of molecular diagnostics. Many recommendations of the “Consortium to Inform Molecular and Practical Approaches to CNS Tumor Taxonomy” (cIMPACT-NOW) have been included [2, 3]. IDH-mutant gliomas were first recognized by the WHO as distinct entities in 2016. In brief, after their initial discovery in glioblastomas arising from lower-grade precursor lesions, so called “secondary” glioblastomas in 2008 [4], it became rapidly evident that IDH-mutations are typical for diffuse lower grade astrocytomas and oligodendrogliomas [5, 6] and associate with a far better prognosis than diffuse IDH-wildtype gliomas [7] most frequently resembling glioblastomas, IDH-wildtype [8]. Furthermore, IDH-mutations invariably present with additional molecular alterations which again are prognostically important: IDH-mutations co-occur either with mutations in *TP53* and *ATRX* associating with a less-favorable prognosis or with whole-arm 1p/19q codeletion and *TERT* promoter (*TERTp*) mutations associating with the most favorable prognosis for IDH-mutant gliomas [9–11]. Because tumors with the trias “IDH/TP53/ATRX” most commonly show an astrocytic differentiation and tumors with the trias “IDH/1p/19q codeletion/TERTp” an oligodendroglial differentiation the designations “astrocytoma” and “oligodendroglioma” were carried on by the WHO to these -by now molecularly defined- tumor types. Specific changes regarding IDH-mutant gliomas in WHO CNS5, unsolved issues and future perspectives will be reviewed and discussed in the following.

## Technical development

Progress in understanding the molecular basis of brain tumors and consequential incorporation of molecular features in brain tumor classification implicates specific needs for molecular analyses. Availability of molecular analyses is fortunately increasing rapidly worldwide. Especially, the influence of DNA-methylation profiling on WHO CNS5 is substantial following the results from many studies clearly demonstrating the enormous potential of genome-wide DNA-methylation profiling for brain tumors classification [12, 13]. Beside providing a methylation profile the method additionally provides information on chromosomal copy number alterations (e.g. for the detection of 1p/19q codeletion). In fact, almost all tumor entities in WHO CNS5 exhibit specific DNA-methylation profiles including astrocytoma, IDH-mutant and oligodendroglioma, IDH-mutant and 1p/19q-codeleted. While large centers already use next-generation sequencing of DNA and RNA as well as genome-wide DNA-methylation profiling for routine diagnostics, it has to be emphasized that most diagnostically relevant molecular alterations may still be determined using more conventional techniques like Sanger sequencing (for *IDH1/2* status determination), fluorescence in situ hybridization (FISH; for 1p/19q and *CDKN2A/B* status determination) or immunohistochemistry (for IDH1 R132H- and ATRX-testing).

## Revised taxonomy and nomenclature

The novel WHO classification aimed for simplification, standardization and harmonization with classifications from other organ systems. While in the 2016 update IDH-mutant tumors comprised 5 different entities (Diffuse astrocytoma WHO grade II, IDH-mutant; Anaplastic astrocytoma WHO grade III, IDH-mutant; Glioblastoma WHO grade IV, IDH-mutant; Oligodendroglioma WHO grade II, IDH-mutant and 1p/19q-codeleted; Anaplastic oligodendroglioma WHO grade III, IDH-mutant and 1p/19q-codeleted) the novel classification recognizes just two entities, now termed “types”: “Astrocytoma, IDH-mutant” and “oligodendroglioma, IDH-mutant and 1p/19q-codeleted”. Grading is performed *within* these types and Arabic instead of Roman numerals are used. To avoid confusion with grading systems from other organs, the term “CNS WHO grade” is endorsed. The range of grades for astrocytomas (grades 2, 3 or 4) and oligodendrogliomas (grades 2 and 3) remains the same. Of note, the term “glioblastoma” is persevered for IDH-wildtype tumors only and the former “glioblastoma, IDH-mutant” has been renamed “Astrocytoma, IDH-mutant, CNS WHO grade 4”. Additional terms like “diffuse” or “anaplastic” are not used anymore (Table 1). To emphasize the biological differences of brain tumors typically occurring in different age groups, CNS5 features a grouping of IDH-mutant gliomas together with glioblastomas, IDH-wildtype as “adult-type” diffuse gliomas, separated from “pediatric type” low- and high-grade diffuse gliomas, even though IDH-mutant gliomas can occasionally develop in the pediatric population as well.

**Table 1:** Terminology of IDH-mutant gliomas in the WHO classification of 2016 and 2021

WHO 2016	WHO 2021
Diffuse Astrocytoma WHO grade II, IDH-mutant	Astrocytoma, IDH-mutant CNS WHO grade 2
Anaplastic Astrocytoma WHO grade III, IDH-mutant	Astrocytoma, IDH-mutant CNS WHO grade 3
Glioblastoma WHO grade IV, IDH-mutant	Astrocytoma, IDH-mutant CNS WHO grade 4
Oligodendroglioma WHO grade II, IDH-mutant and 1p/19q-codeleted WHO grade II	Oligodendroglioma, IDH-mutant and 1p/19q- codeleted CNS WHO grade 2
Anaplastic Oligodendroglioma WHO grade II, IDH-mutant and 1p/19q-codeleted WHO grade III	Oligodendroglioma, IDH-mutant and 1p/19q- codeleted CNS WHO grade 3

## Layered diagnostics, integrated diagnosis and NOS diagnoses

Introduced in the 2016 update of the WHO classification the concept of layered and integrated diagnostics is further emphasized in WHO CNS5. Diagnostics should include different layers, namely histology, WHO grading and molecular findings. The WHO conform integrated diagnosis is based on combining histological and molecular information. Respective reports should include all information, including the integrated diagnosis and the different layers it is based on. A not-otherwise-specified (NOS) diagnosis indicates that a molecular workup for a WHO conform integrated diagnoses is not available or has failed, and therefore the diagnosis is purely based on histological assessment [14]. For the first time molecular features are not only relevant for the classification of IDH-mutant gliomas but may impact grading as well.

## Essential and desirable diagnostic criteria

WHO CNS5 provides essential criteria which must be present to make an integrated diagnoses and desirable criteria which clearly support a diagnosis but are not mandatory. The histological finding of “a diffusely infiltrating glioma” and the molecular finding of an *IDH1/2* hotspot mutation are essential for classifying both astrocytoma and oligodendroglioma. Of note, for oligodendroglioma, the IDH-mutation analysis may not be required when DNA methylome profiling is performed and unequivocally assigns the tumor to the methylation class “oligodendroglioma, IDH-mutant and 1p/19q- codeleted”. The diagnosis of astrocytoma requires either the immunohistochemical loss of nuclear ATRX expression or detection of a pathogenic *ATRX* mutation or the exclusion of combined whole-arm deletions of 1p and 19q. Presence of a *TP53* mutation or strong nuclear expression of p53 in > 10% of tumor cells is a desirable finding as well as a methylation profile of “astrocytoma, IDH-mutant” and an astrocytic differentiation by morphology. Diagnosis of “oligodendroglioma, IDH-mutant and 1p/19q-codeleted” requires the detection of combined whole-arm deletions of 1p and 19q. A DNA methylome profile of oligodendroglioma, IDH-mutant and 1p/19q-codeleted, a retained nuclear expression of ATRX and a *TERT* promoter mutation are desirable criteria (Table 2).

**Table 2:** Essential and desirable criteria for the diagnosis of IDH-mutant gliomas

Tumor type	Essential criteria	Desirable criteria	Additional altered genes
Astrocytoma, IDH-mutant	Diffusely infiltrating glioma	Astrocytic differentiation by morphology	*CDKN2A, CDKN2B, CDK4, CCND2, PDGFRA, MET, MYC, MYCN, RB1, PIK3R1, PIC3CA, others*
	*IDH1/2* hotspot mutation	*TP53* mutation or strong nuclear expression of p53 in > 10% of tumor cells	
	ATRX loss or *ATRX* mutation OR exclusion of combined whole-arm deletions of 1p and 19q	Methylation profile of astrocytoma, IDH-mutant	
Oligodendroglioma, IDH-mutant and 1p/19q-codeleted	Diffusely infiltrating glioma	Retained nuclear expression of ATRX	*CDKN2A, CDKN2B, CIC, FUBP1, NOTCH1*
	*IDH1/2* hotspot mutation	*TERT* promoter mutation	
	Combined whole-arm deletions of 1p and 19q	DNA methylome profile of oligodendroglioma, IDH-mutant and 1p/19q-codeleted	

## Dual-genotype and NEC diagnoses

Not elsewhere classified (NEC) diagnoses are reserved for situations in which all appropriate information regarding e.g. histology, *IDH1/2*-mutation, ATRX and 1p/19q status are available, but results do not match a WHO diagnosis or are conflicting regarding WHO essential criteria [14]. In respect to IDH-mutant gliomas this is of relevance for so called “dual-genotype” IDH-mutant gliomas. These are exceedingly rare and characterized by the presence of defining molecular alterations of both astrocytoma (ATRX loss/mutation and *TP53* mutation) and oligodendroglioma (whole-arm 1p/19q codeletion). These features have been found either in two morphological distinct regions of the same tumor mass or as a single clone in all tumor regions [15–17]. As these cases fall out of the current WHO classification principle, a layered diagnosis providing all histological and molecular information with the addition of “NEC” is suggested.

## Grading of astrocytoma

Grading of astrocytoma IDH-mutant in WHO CNS5 on the one hand retains classic morphological features for grading but on the other hand includes for the first time a molecular feature, namely the homozygous deletion of the *CDKN2A* and/or *CDKN2B* locus. *CDKN2A* and/or *CDKN2B* homozygous deletions have been shown to associate with a dismal prognosis consistently in multiple studies, justifying a grade 4 designation of affected tumors irrespective of the presence of absence of additional features [18]. *CDKN2A* and/or *CDKN2B* homozygous deletions are therefore by definition absent in grade 2 and grade 3 tumors but do enforce grade 4.

**Fig. 1:**
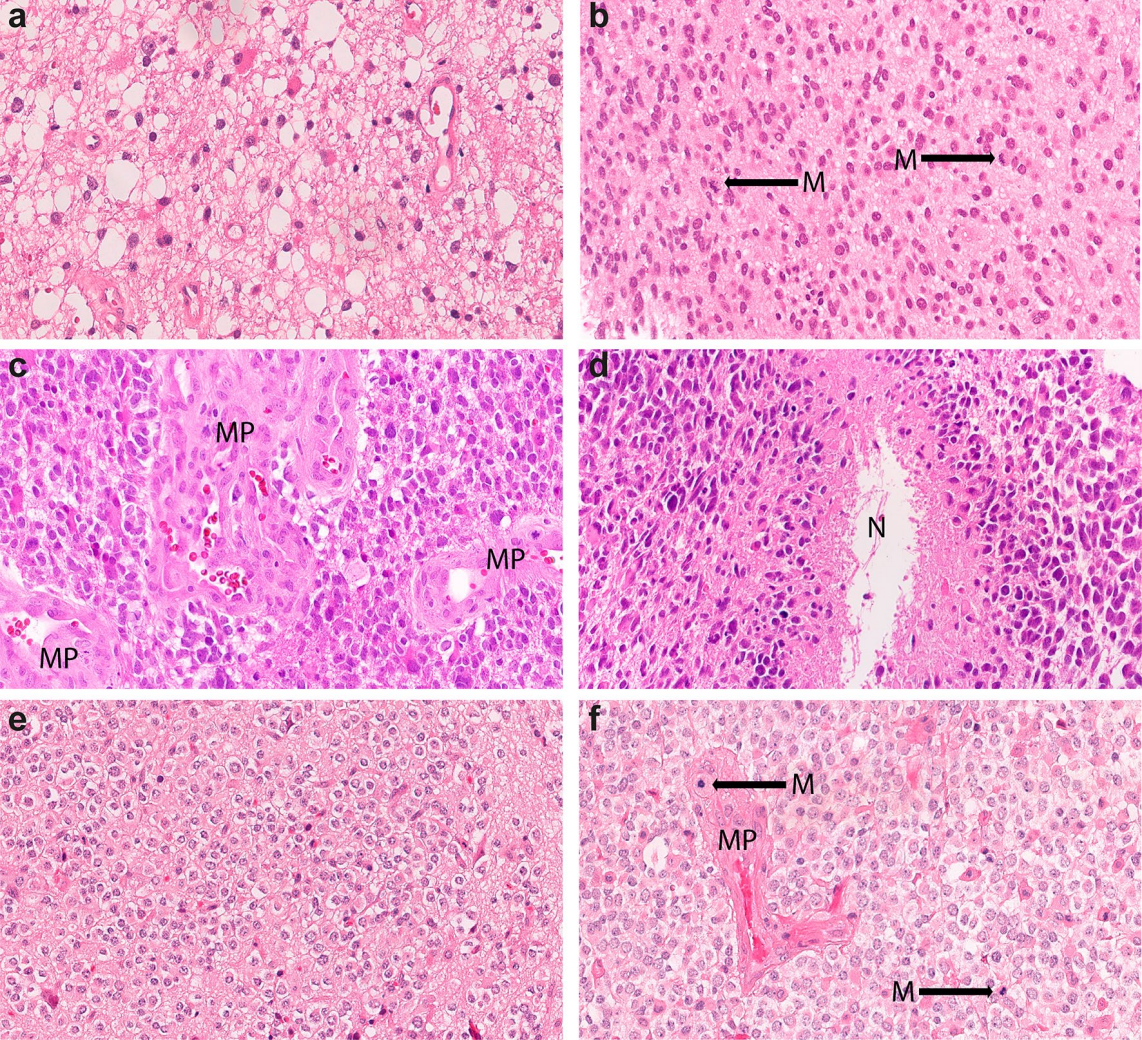
Histology of IDH-mutant gliomas. Astrocytoma, IDH-mutant CNS WHO grade 2 with low cellularity and mild nuclear atypia (**a**), grade 3 showing increased cellularity and mitotic activity (M, arrows) (**b**), grade 4 exhibiting microvascular proliferation (**c**, MP) and palisading necrosis (**d**, N). Oligodendroglioma, IDH-mutant and 1p/19q-codeleted CNS WHO grade 2 displaying the typical honeycomb appearance (**e**) and grade 3 showing microvascular proliferation (MP) and increased mitotic activity (M)

**Fig. 2:**
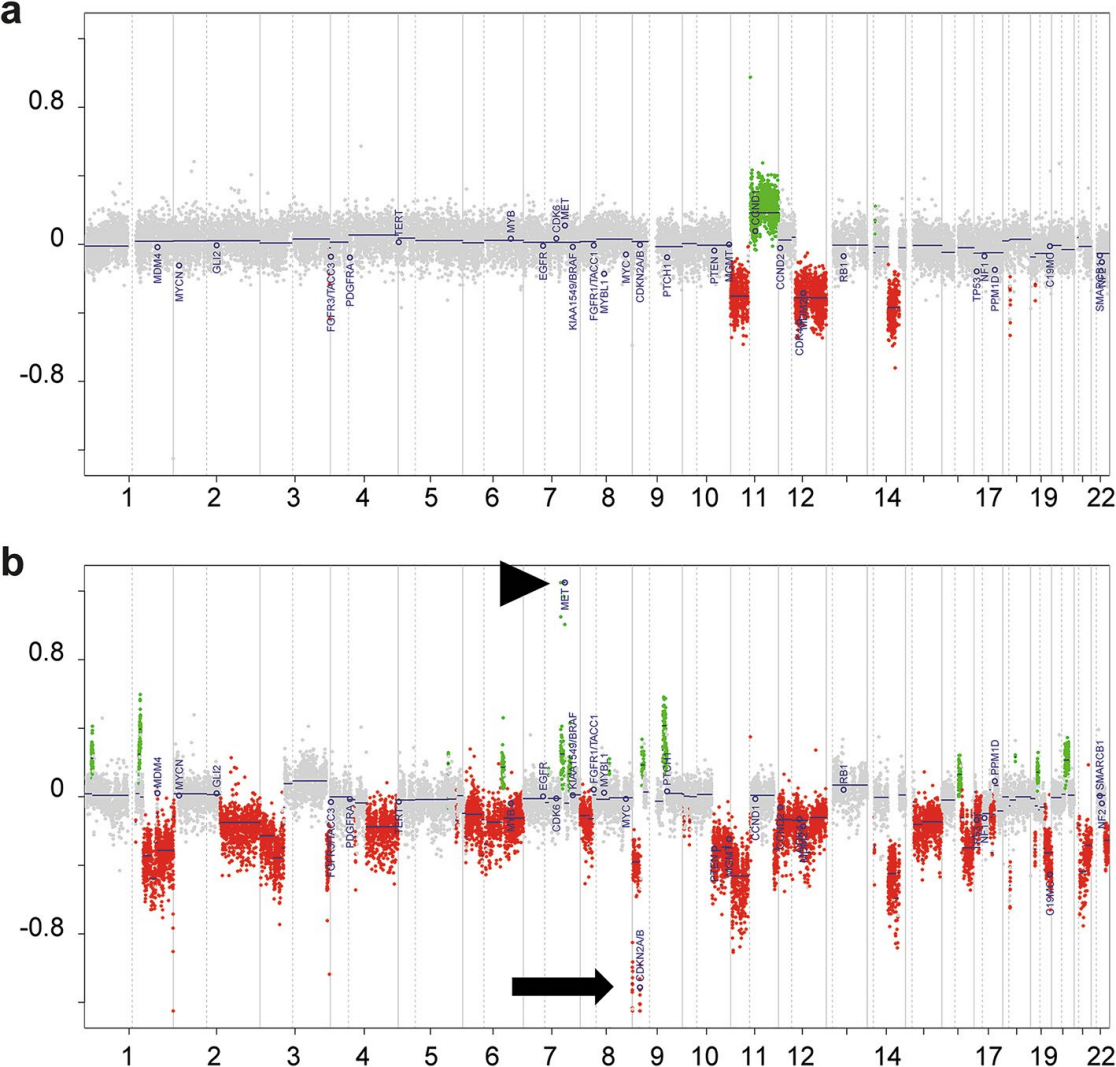
Chromosomal copy number plots from an astrocytoma, IDH-mutant at its primary manifestation corresponding to CNS WHO grade 2 (**a**) and at recurrence, corresponding to CNS WHO grade 4 (**b**). Note the overall increase of chromosomal copy number alterations (gains marked green and losses marked red) in the recurrent tumor including a homozygous *CDKN2A/B* (arrow) deletion and a *MET* amplification (arrow-head)

CNS WHO grade 2 tumors represent the lower end of the malignancy spectrum, showing well-developed differentiation, low to moderate cell density, mild nuclear irregularities and no or very low mitotic activity. Criteria for CNS WHO grade 3 tumors are “focal or dispersed anaplasia” and “significant mitotic activity” while microvascular proliferation, necrosis, and homozygous deletions of *CDKN2A* and/or *CDKN2B* are by definition absent (Fig. 1). Anaplasia manifests in increased cell density, increased nuclear atypia, multinucleation and abnormal mitosis. Cutoff values or thresholds for both mitotic activity and anaplasia are not established with the exception that a single mitosis in a very small biopsy (like a stereotactic biopsy) may suffice for a grade 3 designation. CNS WHO grade 4 tumors represent the high end of the malignancy spectrum and are defined by the presence of at least one of the following features: microvascular proliferation, necrosis or homozygous deletion of *CDKN2A* and/or *CDKN2B* (Table 3, Figs. 1, 2).

**Table 3:** Grading criteria for astrocytomas, IDH-mutant

CNS WHO grade	Criteria
2	Well differentiated and lacks histological features of anaplasia
	Mitotic activity is not detected or very low
	Microvascular proliferation, necrosis, and homozygous deletions of *CDKN2A* and/or *CDKN2B* are absent
3	Focal or dispersed anaplasia
	Significant mitotic activity
	Microvascular proliferation, necrosis, and homozygous deletions of *CDKN2A* and/or *CDKN2B* are absent
4	Microvascular proliferation, necrosis or homozygous deletion of *CDKN2A* and/or *CDKN2B*

## Grading of oligodendroglioma

Grading of oligodendroglioma, IDH-mutant and 1p/19q-codeleted is mainly based on classic histological features of malignancy, such as cellularity, cytological atypia, mitotic activity, microvascular proliferation and necrosis (Fig. 1). WHO CNS5 does not provide cutoffs for parameters and the impact of individual features remains unclear. Microvascular proliferation and brisk mitotic activity (defined as ≥ 2.5 mitoses/mm^2^; equating to ≥ 6 mitoses/10 HPF of 0.55 mm in diameter and 0.24 mm^2^ in area) have been suggested as significant prognostic indicators in some studies while in others microvascular proliferation (with or without necrosis) was more strongly associated with shorter survival than mitotic activity [19–21]. In borderline cases, WHO CNS5 endorses consideration of additional features like extend of KI-67 labeling or neuroradiological features like rapid tumor growth. Cut-off values or thresholds for these criteria are however not established. A novelty is that homozygous deletion involving the *CDKN2A* and/or *CDKN2B* locus is of relevance for the grading of oligodendroglioma as well. Presence of a homozygous *CDKN2A* and/or *CDKN2B* deletion indicates a WHO CNS grade 3. Testing may be restricted to borderline cases as homozygous *CDKN2A* and/or *CDKN2B* deletions have been found in a subset of grade 3 tumors only [22].

## Developments beyond WHO CNS5

The current classification defines two types, astrocytoma and oligodendroglioma, with different grades being listed as subtypes. Recently, several studies some of which were published during the final stage of writing CNS5 or after the completion of CNS5, suggest the existence of clinically relevant (sub-)types of IDH-mutant gliomas. These may have the potential to influence future WHO classifications. Infratentorial IDH-mutant astrocytomas display several characteristics important for diagnostics, which are already mentioned in CNS5. In contrast to supratentorial IDH-mutant astrocytomas, infratentorial astrocytomas frequently harbor non-IDH1 R132H mutations such as IDH1 R132C/G and IDH2 R172S/G limiting the sensitivity of IDH1 R132H immunohistochemistry. The frequency of ATRX loss is also reduced in these tumors increasing the need for molecular testing for IDH-mutation and 1p/19q status. Clinical outcome of infratentorial IDH-mutant astrocytoma was notably worse than that of supratentorial tumors [23]. CNS5 already states that IDH-mutant tumors may occur in the setting of DNA mismatch repair deficiency syndromes and that the clinical management of respective tumors may differ from conventional sporadic cases [24, 25]. The most recent post-CNS5 study established a very poor prognosis for these tumors, comparable with that of IDH-wildtype glioblastomas. The name “Primary mismatch repair deficient IDH-mutant astrocytoma (PMMRDIA)” was proposed to underline their distinctiveness in pathogenesis and clinical behavior [26]. PMMRDIAs have a hypermutant genotype, often paired with microsatellite instability, an altered DNA-methylation profile and show frequent genetic inactivation of RB1- and activation of RTK/PI3K/AKT pathways. PMMRDIAs more likely occur in children and young adolescents. In CNS5 sarcomatous differentiation is described as a rare finding in grade 3 oligodendrogliomas. A post-CNS5 study suggests “oligosarcoma, IDH-mutant” as highly distinct from conventional oligodendroglioma on multiple levels including histology, epigenetic-, proteomic- and molecular profiles. Clinical outcome was significantly poorer for oligosarcomas than for grade 3 oligodendrogliomas [27].

## Conclusion, discussion and outlook

WHO CNS5 classification moves forward towards a classification which is based on tumor biology and serves clinical needs. New grading criteria implicate that the diagnoses of an IDH-mutant glioma which dates back many years may not be in accordance with the novel classification anymore. This should be considered for patient care and is also of importance for retrospective studies.

There is a broad consensus that grading of IDH-mutant gliomas is reasonable given the obvious diversity regarding biological aggressiveness and clinical outcome ranging from patients surviving decades to those which succumb to disease within a few years. However, it is clear that traditional grading criteria perform less well in molecularly defined tumor cohorts due to higher homogeneity. Thus, the separation of grade 2 from grade 3 astrocytomas is controversial. This is mainly due to the fact that glioblastomas, IDH-wildtype, lacking the otherwise typical histological features of microvascular proliferation and/or necrosis, were formerly annotated as anaplastic astrocytomas WHO grade III. Removing these from respective tumor cohorts revealed astrocytomas, IDH-mutant of grades 2 and 3 to display a much smaller differences in survival than previously expected. Studies on grading of diffuse astrocytic gliomas were comprehensively reviewed elsewhere [28]. In fact, while some studies still found statically significant differences others did not [29–33]. In addition, several studies aiming at defining cut-off values e.g. for proliferation or mitotic activity failed or provided inconsistent results [31, 32, 34, 35]. Including homozygous *CDKN2A* and/or *CDKN2B* deletions as grading criteria will increase the fraction of grade 4 tumors but is not expected to substantially alter the decision making for borderline cases between grade 2 and 3. Therefore, room for further improvement remains. There are several additional molecular alterations known to occur in IDH-mutant gliomas during tumor progression: e.g. amplifications of *CDK4*, *PDGFRA*, *MET*, *MYCN*, homozygous deletions of *RB1*, deletions of 14q, mutations in *PIK3R1 or PIC3CA* as well as a higher global copy number variation load [30, 31, 36–41]. Determination of the potential individual impact of these features on prognosis is complex because many of these occur concomitantly. Their prognostic value remains not as well established as for homozygous *CDKN2A* and/or *CDKN2B* deletions and currently these alterations are not of relevance for WHO grading. Additional insights into the process of tumor progression and the development of therapeutic resistance as well as more data from large molecularly and clinically well characterized cohorts will be necessary to further improve classification and grading of IDH-mutant gliomas.
